# Association between angiotensin receptor blocker use and postmortem dementia pathology: analysis of the UK Brain Banks Network dataset

**DOI:** 10.1136/bmjno-2025-001342

**Published:** 2026-04-09

**Authors:** Muzuki Ueda, Daniel Erskine, Alan Thomas, Calum Hamilton, Paul C Donaghy

**Affiliations:** 1 NIHR Newcastle Biomedical Research Centre, Newcastle University, Newcastle upon Tyne, UK

**Keywords:** ALZHEIMER'S DISEASE, COGNITION, DEMENTIA, LEWY BODY DEMENTIA, NEUROPATHOLOGY

## Abstract

**Background:**

Angiotensin receptor blockers (ARB) may be associated with a lower risk of a clinical dementia diagnosis. This study investigated the association between ARB use during life and postmortem dementia neuropathology, compared with angiotensin-converting enzyme inhibitors (ACEI) use.

**Method:**

Cases with documented ACEI (n=257) or ARB (n=102) use, regardless of dementia pathology, were selected from the UK Brain Banks Network dataset. Pathology was categorised as either ‘significant’ pathology (Thal amyloid phase >3, Braak neurofibrillary tangle stage >3, Consortium to Establish a Registry for Alzheimer’s Disease (CERAD) score of moderate/high density and Lewy body (LB) Braak >0 or cortical/limbic/neocortical LB pathology) or ‘not significant’.

**Results:**

The ARB group was less likely to have Alzheimer’s disease (AD) pathology compared with the ACEI group: Thal amyloid (adjusted OR (AOR) 0.59 (95% CI 0.36 to 0.97), p=0.038), Braak neurofibrillary tangle (AOR 0.61 (95% CI 0.38 to 0.98), p=0.041) and CERAD neuritic plaque (AOR 0.58 (95% CI 0.35 to 0.96), p=0.035). LB pathology did not differ significantly between ARB and ACEI groups, though use of either ACEI or ARB was associated with a lower likelihood of LB pathology (AOR 0.27 (95% CI 0.21 to 0.36), p<0.001).

**Conclusion:**

ARB use is associated with a lower risk of AD pathology. The association between ARB/ACEI use and LB pathology requires further investigation.

## Background

 The global prevalence of dementia is expected to rise substantially in the coming decades, but available dementia treatments offer limited efficacy and are expensive to deliver.^1^

Hypertension is a well-established risk factor for dementia.^2^ In the UK, angiotensin-converting enzyme inhibitors (ACEI) and angiotensin receptor blockers (ARB) are equally recommended as first-line treatment for hypertension. Both ACEIs and ARBs modulate the renin–angiotensin system (RAS), a critical regulatory pathway for blood pressure. While both drug classes may contribute to dementia risk reduction through blood pressure control, ARBs may offer unique advantages in neuroprotection by reducing neuroinflammation and oxidative stress, and by increasing acetylcholine release, through potential mechanisms explained by the angiotensin hypothesis.^3^

Recent evidence has demonstrated that ARBs are associated with a lower risk of clinical diagnosis of dementia and Parkinson’s disease.^4 5^ There are only a few neuropathological studies that have studied the effect of ARBs on Alzheimer’s disease (AD) pathology postmortem^6 7^ and there are no studies on its effect on Lewy body (LB) pathology, the second most common type of neurodegenerative pathology associated with dementia.

This study aimed to investigate the relationship between ARB use and dementia neuropathology by comparing the proportion of cases with significant AD and LB pathology among ACEI- and ARB-exposed groups in the UK Brain Banks Network (UKBBN) dataset.

## Method

### Subjects

The UKBBN dataset includes data from over 15 000 brain donors recruited from 10 brain banks in the UK.^8^ The donors include both people with and without dementia. Medical history was collected from participant/carer reports and medical records where available.

Neuropathologists at each site assigned Thal amyloid phases for amyloid-β deposition, CERAD scores for neuritic plaque density and Braak neurofibrillary tangle stage for neurofibrillary tangle presence.^9^ LB neuropathology was classified according to Braak LB stages and Lewy pathology consensus criteria.^10^

### Data extraction

All cases with a record of ACEI and/or ARB use were identified in the UKBBN dataset (downloaded on 7 February 2024). Cases on both ACEI and ARB, cases aged under 50 and cases lacking neuropathological data were excluded from the study. Baseline characteristics, including age at death, sex, baseline clinical dementia rating (CDR) score and apolipoprotein e4 allele (APOE e4) carrier status, were recorded.

### Statistical analysis

Each neuropathological staging system was categorised as either ‘significant’ pathology (Thal amyloid phase >3, Braak neurofibrillary tangle stage >3 and Consortium to Establish a Registry for Alzheimer’s Disease (CERAD) neuritic plaque score of moderate/high density) or ‘not significant’. LB pathology was categorised as ‘significant’ if Braak LB stage >0 or if Lewy pathology consensus criteria was brainstem, limbic or neocortical.

Thresholds of significant pathology were determined based on the stages likely to be clinically significant and the distribution of pathology across the dataset, prior to analysing the medication data.

The association between ACEI or ARB use and significant pathology in each neuropathological staging system was evaluated using a binary logistic regression model, adjusting for age at death and sex.

Covariates, including baseline CDR, were examined in sensitivity analyses due to incomplete data availability across cases.

All analyses were performed using the SPSS Statistics 30 software.

## Results

Of the 413 cases with a history of ACEI and/or ARB use, cases on both ACEI and ARB (n=22), cases with age at death under 50 years (n=4) and cases with no neuropathological data (n=28) were excluded. Analysis was performed on the remaining cases (n=359), which consisted of cases only on ACEIs (n=257) and cases only on ARBs (n=102). The combined ACEI or ARB group was also compared with those on neither medication (n=6641). Characteristics of the entire UKBBN cohort are summarised in online supplemental table 1.

Sex distribution, age at death, baseline CDR score and APOE e4 status were similar in the ARB and ACEI groups.

The ARB group had lower rates of significant AD pathology compared with the ACEI group for Thal amyloid (40.2% vs 52.5%, adjusted OR (AOR) 0.59 (95% CI 0.36 to 0.97), p=0.038), Braak neurofibrillary tangle (35% vs 47%, AOR 0.61 (95% CI 0.38 to 0.98), p=0.041) and CERAD neuritic plaque (33% vs 45.6%, AOR 0.58 (95% CI 0.35 to 0.96), p=0.035). A smaller proportion of cases in the ARB group had significant LB pathology compared with the ACEI group (20.9% vs 26.2%), but the difference was not statistically significant (AOR 0.74 (95% CI 0.41 to 1.30), p=0.315). Sensitivity analysis using baseline CDR score yielded similar adjusted ORs to the primary analysis, despite reduced significance from a smaller sample size (see online supplemental table 2).

When comparing the ‘ACEI or ARB’ group to the ‘No ACEI/ARB’ group, a significant reduction was observed specifically for LB pathology (24.6% vs 53.6%, AOR 0.27 (95% CI 0.21 to 0.36), p<0.001).

## Discussion

This study explored the association between ARB use and postmortem dementia neuropathology compared with ACEI use within the UKBBN dataset.

### Key findings

A lower percentage of significant pathology cases was found in the ARB group compared with the ACEI group across all neuropathological staging systems, with statistically significant differences in AD pathology but not in LB pathology.

### ARB and AD pathology

Observational studies have consistently demonstrated that ARB use is associated with reduced dementia risk compared with other antihypertensive classes.^4^ However, there are limited neuropathological studies that demonstrate a link between ARB use and key dementia pathologies in humans, highlighting the importance of our study.

Previous neuropathological studies examining the association between antihypertensive use and dementia have used the National Alzheimer Coordinating Center (NACC) database. A study published in 2012 using the NACC database (n=890) found that ARB use was associated with a statistically significant reduction in CERAD neuritic plaque burden and Braak neurofibrillary tangle stages compared with other antihypertensives.^6^ This study excluded participants with normal cognition.^6^ Our findings provide further support that ARB use could be associated with lower rates of AD pathology.

Preclinical evidence suggests that ARB may have benefits beyond blood pressure regulation in preventing AD by selectively blocking proinflammatory AT1 receptors from binding to angiotensin II, thereby reducing amyloid-β generation and accumulation.^3^

### ARB and LB pathology

LB pathology is associated with dementia with Lewy bodies (DLB) and Parkinson’s disease (with or without dementia). No studies have investigated the relationship between ARB use and postmortem LB pathology.

There are no cohort studies on the relationship between ARB use and DLB. Yang *et al* reported that ARB use, but not ACEI, was associated with a slower rate of cognitive decline in patients with Parkinson’s disease and hypertension, based on the Parkinson’s Progress Marker Initiative dataset.^5^ A drug-wide association study using the Norwegian Prescription Registry found that both ARB and ACEI were associated with a decreased risk of Parkinson’s disease to a similar degree (hazard ratio(HR) 0.93 and HR 0.92, respectively).^11^

A low threshold was adopted for LB pathology. While AD pathology is frequently found in cognitively normal elderly individuals, incidental LB pathology is relatively uncommon and is considered a presymptomatic stage of DLB or Parkinson’s disease. Our threshold choice is supported by evidence that incidental LB cases exhibit dopaminergic depletion and regional cell loss intermediate between controls and DLB, indicating an active disease process rather than normal ageing.^12^

Notably, a significantly lower prevalence of LB pathology was observed in the combined ‘ACEI or ARB’ group compared with the ‘No ACEI or ARB’ group. This finding requires cautious interpretation. LB disease often presents with autonomic dysfunction and orthostatic hypotension, which may deter the prescription of antihypertensives. Despite the potential for reverse causality, these findings provide a strong rationale for further investigations into whether the RAS plays a neuroprotective role in LB pathology.

### Strengths and limitations

A major strength is our use of neuropathological outcomes, which are more accurate than clinical diagnoses. By including cases regardless of dementia diagnosis, we captured cases where ARBs may have prevented the onset of dementia altogether. Since ARBs and ACEIs have comparable efficacy in treating hypertension,^13^ this study design enabled investigation of their effects on neuropathology while reducing the impact of hypertension as a confounder.

A limitation was the incomplete data regarding medication dosage, duration and adherence, which precluded their inclusion in the analysis. Low essential hypertension prevalence in the UKBBN likely reflects under-reporting. Additionally, the dataset may over-represent individuals with genetic predispositions to dementia, as indicated by the proportion of APOE e4 carrier cases in table 1, which is much higher than in the general population.^14^ Genetic predisposition could have masked the benefits of ARBs, as previous research has indicated that ARBs were only beneficial in the absence of APOE e4.^15^ In our study, the subset of cases with known APOE e4 non-carrier status (54%) was too small to conduct sensitivity analyses.

**Table 1:** Comparison of cases not receiving ACEI/ARB and those receiving ACEI or ARB

Characteristics	No ACEI/ARB	ACEI or ARB	ACEI	ARB
n	6641	359	257	102
Age, median (IQR)	80 (14)	85 (11.5)	85 (12)	85.5 (12)
Sex, % female	46.1	44.8	44.0	47.1
Baseline CDR, mean (SD)	1.01 (1.20)	0.67 (0.92)	0.71 (0.95)	0.59 (0.87)
Baseline CDR months to death, median (IQR)	61 (79.5)	64.5 (51.25)	61 (55)	66 (43.5)
APOE e4 carrier, n (%)	925/1924 (48.1)	95/194 (49.0)	69/144 (47.9)	26/50 (52)
Dementia present, n (%)	2815/6455 (43.6)	162/359 (45.1)	120/256 (46.9)	41/101 (40.6)
Essential hypertension present, n (%)	566/6455 (8.77)	102/359 (28.4)	74/256 (28.9)	28/101 (27.7)
Type 2 diabetes mellitus present, n (%)	189/6455 (2.93)	47/359 (13.1)	32/256 (12.5)	15/101 (14.9)
Ischaemic heart diseases present, n (%)	263/6455 (4.07)	46/359 (12.8)	4/256 (1.56)	1/101 (0.99)
Cerebrovascular disease present, n (%)	407/6455 (6.31)	26/359 (7.24)	19/256 (7.42)	7/101 (6.93)

Neuropathological staging system	No ACEI/ARB	ACEI or ARB	P value	AOR (95% CI)	ACEI	ARB	P value	AOR (95% CI)
Thal amyloid >3, n (%)	1384/3293 (42.0)	154/315 (48.9)	0.159	1.18 (0.94 to 1.50)	117/223 (52.5)	37/92 (40.2)	0.038	0.59 (0.36 to 0.97)
Braak neurofibrillary tangle >3, n (%)	2717/6158 (44.1)	154/353 (43.6)	0.592	0.94 (0.76 to 1.17)	119/253 (47.0)	35/100 (35.0)	0.041	0.61 (0.38 to 0.98)
CERAD neuritic plaque moderate/high density, n (%)	1535/3361 (45.7)	141/335 (42.1)	0.074	0.81 (0.64 to 1.02)	110/241 (45.6)	31/94 (33.0)	0.035	0.58 (0.35 to 0.96)
Lewy body pathology present, n (%)	2220/4138 (53.6)	74/301 (24.6)	<0.001	0.27 (0.21 to 0.36)	55/210 (26.2)	19/91 (20.9)	0.315	0.74 (0.41 to 1.30)

ACEI, angiotensin-converting enzyme inhibitors; AOR, adjusted odds ratio; APOE e4, apolipoprotein e4 allele; ARB, angiotensin receptor blockers; CDR, clinical dementia rating; CERAD, Consortium to Establish a Registry for Alzheimer's Disease.

## Conclusion

This study established a significant association between ARB use and AD pathology, supporting existing evidence on the potential protective effects of ARBs compared with ACEIs. These findings support the ongoing efforts to repurpose ARBs for the prevention of dementia. Further research is required to investigate the relationship between ACEI or ARB use and LB pathology.

## Supplementary material

10.1136/bmjno-2025-001342 online supplemental file 1

## Data Availability

Data are available upon reasonable request.
