# Supplementary material for: Association between angiotensin receptor blocker use and postmortem dementia pathology: analysis of the UK Brain Banks Network dataset
Source: BMJ Neurol Open. 2026 Apr 9;8(1):e001342. doi: 10.1136/bmjno-2025-001342 (PMC13084883; doi:10.1136/bmjno-2025-001342)
Supplement: online supplemental file 1 [file bmjno-8-1-s001.docx]

**Supplementary Table 1. Characteristics of the entire UKBBN Cohort.**

| Characteristics | Entire UKBBN Cohort |
| --- | --- |
| n | 15939 |
| Age, median (IQR) | 74(22) |
| Sex, % female | 45.8 |
| Baseline CDR, mean (SD) | 0.94(1.16) |
| Baseline CDR months to death, median (IQR) | 62(74) |
| APOE e4 carrier, n (%) | 1347/3588(37.5) |
| Dementia present, n (%) | 4351/15056(28.9) |
| Essential hypertension present, n (%) | 864/15056 (5.74) |
| Type 2 diabetes mellitus present, n (%) | 303/15056 (2.01) |
| Ischaemic heart diseases present, n (%) | 437/15056 (2.90) |
| Cerebrovascular disease present, n (%) | 687/15056 (4.56) |

**Supplementary Table 2. Odds of pathology for donors taking ARBs compared with ACEIs, with CDR score as a covariate.**

| Neuropathological staging system | n | ACEI significant pathology | ARB significant pathology | p-value | Adjusted odds ratio (95% CI) |
| --- | --- | --- | --- | --- | --- |
| Thal amyloid >3 | 248 | 88/170(51.8) | 31/78(39.7) | 0.088 | 0.60(0.34-1.08) |
| Braak neurofibrillary tangle >3 | 255 | 84/176 (47.7) | 29/79(36.7) | 0.159 | 0.63(0.33-1.20) |
| CERAD neuritic plaque moderate/high density | 251 | 83/174(47.7) | 27/77(35.1) | 0.113 | 0.61(0.33-1.13) |
| Lewy body pathology present | 241 | 40/166(24.1) | 15/75(20.0) | 0.499 | 0.79(0.40-1.56) |

*Independent variables of the binomial logistic regression are medication (ACEI or ARB), age at death, sex, and baseline Clinical Dementia Rating (CDR) score.*
